# Brief Overview of Bioinformatics Activities in Singapore

**DOI:** 10.1371/journal.pcbi.1000508

**Published:** 2009-09-25

**Authors:** Frank Eisenhaber, Chee-Keong Kwoh, See-Kiong Ng, Wing-King Sung, Limsoon Wong

**Affiliations:** 1 BioInformatics Institute, Singapore; 2 Nanyang Technological University, Singapore; 3 Institute for Infocomm Research, Singapore; 4 Genome Institute of Singapore, Singapore; 5 National University of Singapore, Singapore; University of California San Diego, United States of America

## Introduction

The frontier of biological and medical sciences is full of opportunity today. It is widely appreciated that present-day biomedical researchers are confronted by vast amounts of data from genome sequencing; microscopy; high-throughput analytical techniques for DNA, RNA, and proteins; and a host of other new experimental technologies. Coupled with advances in computing power, this flow of information enables scientists to computationally model and analyze biological systems in novel ways. Therefore, bioinformatics is seen as an important ingredient in Singapore's ambition to be an international center for the biomedical sciences and their related industries.

Five organizations are involved in bioinformatics in Singapore in a major way. Two of these are universities in Singapore, namely the National University of Singapore (NUS) and the Nanyang Technological University (NTU). NUS has a longer history in bioinformatics and life science training and research, while NTU did not have a life science school until the early 2000s. The other three are institutes under the Agency for Science Technology & Research (A*STAR), namely the BioInformatics Institute (BII), the Genome Institute of Singapore (GIS), and the Institute for Infocomm Research (I^2^R). I^2^R has the longest history in this field in Singapore, and it accounted for a lion's share of Singapore's output in bioinformatics research from 1994 to 2005. BII and GIS are entities set up in the early 2000s; they have now matured into major forces in bioinformatics research in Singapore.

An earlier report describes the development and personalities of Singapore bioinformatics from 1992 to 2002 [1]. The bioinformatics scene in Singapore has undergone some important changes since 2005, with new leadership in three of the five major centers of activities in Singapore—BII, I^2^R, and NUS. Here, we provide an updated overview of bioinformatics research and training activities at these organizations, as well as at GIS and NTU.

## Research at BII

BII (http://www.bii.a-star.edu.sg) of A*STAR was originally founded in 2001. After a tumultuous history with changing missions and directors, BII was essentially relaunched in the autumn of 2007. Now its mission is defined primarily as a computational biology research institute. Its new director, Frank Eisenhaber (previously at the Research Institute of Molecular Pathology in Vienna, Austria), guides the transition.

BII sees its future as a center for research in the field of biomolecular mechanism exploration driven by computational biology. Thus, BII is meant to remain primarily a theoretical institute. But in contrast to the previous concept, experimental work has a place at the Institute both for the follow-up of theoretically derived hypotheses and for the generation of datasets that are important for the development of theoretical approaches to biological problems.

The emphasis on biomolecular mechanisms is guided both by fundamental and by pragmatic considerations. Computational biology will have a great impact in this area since the ever-increasing body of sequence data, together with other large-scale datasets on expression, structure, interaction, and subcellular localization of biomolecules, provide great opportunities for achieving new biological insight using theoretical arguments. BII is located in the Biopolis in the Buona Vista area of Singapore and wishes to find synergies by interacting with the community, especially with other A*STAR biomedical research institutes that concentrate on genomics (GIS), molecular and cellular biology (IMCB), as well as their context with human disease (IMB, SiCS, SiGN), and with biotechnology applications (BTI, ETC, IBN).

At present, BII hosts 11 independent research teams organized into four research divisions. The “Imaging Informatics” section develops automated tools for the quantification of the distribution of labeled molecules with regard to subcellular structures in images of cells. BII's own microscopy lab is coming into operation in summer 2009. In the “Genome Sequence and Gene Expression Data Analysis” division organized by Vladimir Kuznetsov, the research focus is on understanding transcriptional regulation and the biological role of non-coding RNA. Chandra Verma guides the “Biomolecular Structure and Design” division, the teams of which analyze and simulate 3D structural assemblies of biomolecules and try to connect structural features with biological function.

Finally, the “Biomolecular Function Discovery” unit is quite a unique setup since it combines a protein sequence analysis group with a biochemical laboratory for the verification of predicted gene functions and a software team working on the ANNOTATOR environment, a system of workflows for annotating uncharacterized protein sequences.

Given that any really serious scientific project takes a few years, time will tell whether the promise of BII will be realized. Nevertheless, several recent publications show a glimpse of BII's opportunities. For example, the mutations of the neuraminidase from the 2009 H1N1 (swine flu) virus strain have been shown not to affect the binding pocket of the antiviral drugs oseltamivir (Tamiflu), zanamivir (Relenza), and peramivir [2]. As another example, the ANNIE software sets a new standard in protein sequence annotation and function prediction [3].

BII offers the opportunity for Ph.D. students who are affiliated with any university in the world (for their examinations and their degree) to carry out research on one of the teams and to receive local monetary support over a period of three years.

## Research at I^2^R

I^2^R (http://www.i2r.a-star.edu.sg) of A*STAR is a research institute for information technologies. As such, it devotes a small amount of its resources to bioinformatics, namely part of its data mining department. The primary objective of the bioinformatics research program at I^2^R is to inspire new research in data mining through computational analysis of biomedical data. Since See-Kiong Ng took over as manager of the data mining department in 2006, the group has focused on two areas, namely text mining and graph mining, to address the computational challenges brought about by the abundance of unstructured text and interaction networks in biology.

As one of the early pioneers in biomedical text mining [4], I^2^R has been developing effective text-mining approaches for extracting useful information from the vast biomedical literature. The group actively participates in international efforts in this domain. For example, they are part of the EU's BOOTStrep (Bootstrapping Of Ontologies and Terminologies STrategic REsearch Project) program to develop an integrated text analysis system for biological documents, and they also collaborate with Tokyo University in developing a large-scale co-reference corpus on Medline abstracts. The group's text-mining methods have been shown to be among the best in international benchmark competitions such as BioCreAtIvE [5].

For graph mining, the group has been focusing on the analysis of whole-genome protein–protein interaction networks, addressing such practical issues as handling the high abundance of experimental errors in the data and the effective integration of domain knowledge in the analysis. Given that biological systems are largely made up of networks of molecular interactions, developing data-mining methods to discover useful patterns from large networks is essential for understanding how cellular biology works, even though graph mining is intrinsically challenging computationally, with many problems proven to be NP-hard. The group collaborates extensively with local universities to develop algorithms that can be applied to experimentally determined protein–protein interaction networks to discover new biological knowledge such as domain interactions [6] and protein complexes [7].

I^2^R also has an emphasis on applied research. As such, the group is driven by the need to apply the computational methods developed to help biologists deepen their understanding of molecular biology, and to harvest the knowledge gained to combat the many health threats that Singapore faces today. One unique biological application domain that the bioinformatics group at I^2^R has been focusing on is computational immunology. This is particularly relevant to Singapore given its recent close shaves with the SARS and avian flu viruses, as well as the emergence of tropical infectious diseases such as Dengue and Chikungunya fevers. With the alarming increase in worldwide outbreaks in the last few years, it is clearly also of great global concern. As vaccination has been one of the most successful public health intervention measures against infectious diseases, to gain a fighting chance against these new health threats it is crucial to significantly accelerate the development of vaccines. The group at I^2^R was one of the first to realize that the recent advances in genomic, proteomic, and bioinformatics technologies have offered new opportunities to do so. They have been developing and applying computational methods to screen large sets of protein antigens, such as those encoded by complete viral genomes [8], and validating their computational results by working closely with bench biologists both locally and internationally. Thus far, the group has worked on various viruses such as Dengue, West Nile virus, Yellow Fever virus, Human Influenza A, and Chikungunya. In 2008, the current principal investigator of the project, Joo Chuan Tong, was selected as one of the 35 top innovators in science and technology under the age of 35 by MIT's *Technology Review* magazine for his research in “personalized vaccine design”.

## Research at GIS

GIS (http://www.gis.a-star.edu.sg) is an A*STAR institute focused on genomic research. GIS aims to have a deeper understanding of cancer biology, stem cell biology, molecular pharmacology, and infectious disease through genomic study. Bioinformatics is used as a tool to support the associated high-throughput genomic analyses. Roughly speaking, the bioinformatics work at GIS can be divided into three domains: sequence analysis, comparative genomics, and microarray study.

GIS has developed a series of high-throughput DNA sequencing technologies based on paired-end ditags (PET). These technologies accelerate the understanding of the dynamics and the structure of DNA elements in our complex genome. A computational sequence analysis pipeline is a main vehicle for transforming raw sequencing data into meaningful information. Combined with upstream bioinformatics analysis, it leads to biological discovery. One example is genome-wide fusion gene identification using GIS-PET [9]. In GIS-PET analysis, PETs from the two ends of each expressed transcript (18 bp from the 5′ end and 18 bp from the 3′ end) are extracted. Mapping the PETs onto the reference genome gives the precise transcript boundaries. However, 4%–5% of PETs still cannot be mapped. These PETs may represent unconventional transcripts such as fusion genes whose 5′ and 3′ ends may map on different chromosomes. Through a novel clustering algorithm, 170 fusion gene candidates are identified.

Comparative genomic analysis is applied at GIS to understand the genome rearrangement in cancer and the evolution of regulatory sequences in our genome. For example, analyzing several transcription factors using ChIP-Seq technology [10] showed that a large portion of binding sites are embedded in repeats. More precisely, those binding sites are located in distinctive families of transposable elements. This study indicates that transposable elements play an important role in expanding the repertoire of binding sites.

Microarray analysis is performed daily at GIS for studying gene expression and for diagnosis. In addition to routine bioinformatics analysis, the groups at GIS also develop new technology using the microarray platform. For instance, they developed the pathogen chip [11], which detects the presence of viruses from patient samples in an unbiased manner. The major difficulty for virus detection is how to amplify the complete genomes of the viruses. Researchers at GIS proposed a computational method that designs a random primer that can amplify a selected set of viruses efficiently.

In the future, bioinformatics will remain a main weapon at GIS to understand the mechanisms in our genome. Current work includes understanding the chromatin structure, deciphering the histone code and the transcriptome map, and studying genome rearrangement in cancer genomes. All these works rely heavily on bioinformatics.

## Training Program at NTU

There are two main formal bioinformatics training programs in Singapore. The first is a master's program at NTU. The second is a bachelor's program at NUS (see next section). In this section we describe the former, which is modeled after the approach proposed in [12]. The curriculum comprises a set of core bioinformatics courses that build upon the contributing disciplines to present the basic intellectual structure of the field.

The NTU bioinformatics program offers a two-year part-time or one-year full-time training leading to an M.Sc. degree. It is designed for students who have relevant scientific and technical backgrounds (engineering or science degrees). The curriculum provides them with skills for the creation of excellent well-validated methods for solving problems in the domain of bioinformatics and related fields.

The program gives students enough time to learn about tool use and later on tool development. Full-time students must complete six core modules, two elective modules, and a project to graduate, while part-time students may complete additional elective modules instead of the project. The six core modules are: two biology modules; an introductory bioinformatics module, which trains students to be proficient tool users; a statistics module; and two modules on algorithms for bioinformatics, which train students to put together new efficient tools in addition to being able to apply existing tools. After taking all six core modules, the students are expected to be proficient in implementing, improving, and creating new software tools and methods for analyzing and organizing data. Once this core foundation is laid, the students can move on to select more current and diverse topics in bioinformatics such as high-performance computing for bioinformatics and methods and tools for proteomics.

Due to the multidisciplinary nature of the program, the teaching faculty is drawn from the whole range of engineering and science schools at NTU, such as the School of Computer Engineering, the School of Mechanical and Aerospace Engineering, the School of Electrical and Electronic Engineering, the School of Chemical and Biomedical Engineering, the National Institute of Education, and the School of Biological Sciences. Furthermore, there are several adjunct faculty members from GIS, I^2^R, BII, and the National Cancer Centre who contribute significantly in teaching and supervision.

## Research and Training Program at NUS

There are about twenty faculty members at NUS who are involved in research relating to bioinformatics to some extent. Half of them are in the Computational Biology Lab in the Department of Computer Science (CBL, http://www.comp.nus.edu.sg/~cbl), which has been coordinated by Limsoon Wong since 2005. The BioInformatics and Drug Design Group in the Department of Pharmacy (BIDD, http://bidd.nus.edu.sg/group/research.htm), which has been led by Yuzong Chen since 1997, is the second major center of bioinformatics activities at NUS.

Research at CBL leads to fundamental advances in knowledge discovery technologies, database technologies, combinatorial algorithms, and modeling and simulation technologies, as well as in the applications of these technologies to problems in biology and medicine. Research at BIDD has as its main goals development of computer-aided drug design methods and software, development of bioinformatics databases and software, and tool development for and mechanistic study of traditional Chinese medicine.

Some ongoing projects at NUS include the following.

### Gene Expression Analysis

Existing works on gene expression analysis provide insufficient information on the interplay between selected genes. Also, the collection of pathways that can be used, evaluated, and ranked against the observed expression data is limited. Furthermore, a comprehensive set of rules for reasoning about relevant molecular events has not been compiled and formalized. A more advanced integrated framework to provide biologically inspired solutions for these challenges is envisioned in this project [13].

### Protein Complex Prediction

Protein–protein interaction (PPI) data obtained by high-throughput assays contain a high rate of errors. Thus it is desirable to prioritize PPIs detected by such high-throughput assays. Furthermore, PPI networks resulting from these assays are essentially an in vitro scaffold. Further progress in computational analysis techniques and experimental methods is needed to reliably deduce in vivo protein interactions [14], to distinguish between permanent and transient interactions, to distinguish between direct protein binding from membership in the same protein complex [15], and to distinguish protein complexes from functional modules. This project aims to develop a system to process results of high-throughput PPI assays, as well as integrating extensive annotation information, to yield a more informative protein interactome.

### Protein 3D Structure Analysis

The study of proteins from a structural perspective gives more valuable information about their functions. The two main objectives in this project are to develop efficient and effective methods to compare a pair of 3D protein structures [16] and to develop efficient and effective methods to search a database of 3D protein structures [17].

### Functional Element Identification

Protein interactions with DNA and RNA are the primary mechanisms for controlling gene expression. What is needed is a recognition code that maps from the protein sequence to a pattern that describes the family of DNA binding sites—the functional elements. This project develops methods for accurate identification of transcription factor binding sites and also methods for inferring the interactions of transcription factors and other functional elements [18].

### Protein Motion Simulation and Analysis

Many interesting properties of molecular motion are best-characterized statistically by considering an ensemble of motion pathways rather than an individual one. Classic simulation techniques, such as the Monte Carlo method and molecular dynamics, generate individual pathways one at a time and are easily trapped in the local minima of the energy landscape. They are computationally inefficient if applied in a brute-force fashion to deal with many pathways. The project introduces Stochastic Roadmap Simulation, a randomized technique for sampling molecular motion and exploring the kinetics of such motion by examining multiple pathways simultaneously [19].

### Computational Systems Biology

Computational systems biology involves studying cellular functions and its components at varying degrees of granularity. These levels range from the nano-scale molecular structures (atomic level) to entire organs such as heart and lungs (phenotype level). The project focus is mainly on the functional aspects of cellular components, in the form of biopathways. The team hopes to develop a set of tools and modeling methodology to produce accurate models that can be validated and that can be used to predict new phenomena [20].

In terms of training activities, NUS has a bachelor's program in bioinformatics, where science-based students receive a B.Sc. (Bioinformatics) degree and computing-based students receive a B.Comp. (Bioinformatics) degree. Both sets of students share a core set of bioinformatics courses and basic biology and computing courses. The core bioinformatics courses comprise the following chain of three modules: 1) an introductory computational biology module, which focuses on developing the understanding of bioinformatics problems, the key principles for solving a wide range of bioinformatics problems, and the ability to interpret and analyze the output of various tools and algorithms; 2) a module on combinatorial methods in bioinformatics, which introduces students to combinatorial methods used frequently in a range of bioinformatics problems such as motif finding, population genetics, genome annotation, and RNA structure; and 3) a module on knowledge discovery methods in bioinformatics, which introduces students to data-mining algorithms often used in a range of bioinformatics problems such as gene expression profile analysis and gene feature recognition.

After completing the basic courses and the three core modules described above, the program has a number of advanced computational biology courses that can be chosen as electives.

## Concluding Remarks

As early as 1992, there were already bioinformatics activities in Singapore championed by Tin-Wee Tan at NUS. These activities included mirroring of data collections and development of sequence analysis applications. Bioinformatics activities in Singapore took on a deeper research character when Limsoon Wong started work on the Kleisli query system in 1994 [21]. This work generated significant interest from several large international pharmaceutical companies. This helped the Singapore Economic Development Board become convinced to fund, in 1996, a Bioinformatics Center at NUS as a joint collaboration between the activities of Tan and Wong. By 2000, the potential of bioinformatics in modern biomedical research was fully recognized. Therefore, A*STAR initiated significant new funding to encourage and to support research and development in this area. GIS was established as the flagship organization for high-throughput biological research in Singapore. A year later, BII was established to drive both bioinformatics training and research. However, BII drifted in its twin missions. NUS and NTU responded by establishing proper degree programs in bioinformatics in 2003 and 2002, respectively, as well as by establishing more coordinated bioinformatics research programs in the mid-2000s. In 2007, BII was relaunched with research as its primary mission.

Today, the work of bioinformaticists from Singapore are found in journals and at conferences that are purely computer science, purely biology, purely medicine, as well as in the mainstream bioinformatics journals. In fact, despite the small size of her bioinformatics community (<100), Singapore contributed 1.73% of papers published in *Bioinformatics* since 2000. Furthermore, according to SCOPUS, these papers also account for 1.05% of citations to *Bioinformatics* since 2000. These data and the descriptions in the preceding sections show that bioinformatics activities in Singapore have grown in diversity, intensity, and quality.

This healthy growth in research capability and government funding has helped to attract international drug and life sciences companies to Singapore. For example, a significant portion of Eli Lilly's bioinformatics activities is now based at the Lilly Singapore Centre for Drug Discovery. The ease of recruiting well-trained manpower is crucial to attracting and maintaining such industry R&D centers in Singapore. To groom truly world-class Singaporean researchers, it is important that they gain adequate overseas exposure as part of their training. Because of the focus in research and education in Singapore, many of our local graduates are able to find offers for doctorate and post-doctorate positions in top universities and research centers overseas. There are also ample government sponsorships (e.g., A*STAR scholarships) that provide financial support for the local trainees to go overseas for their doctoral and post-doctoral training. Those who take up such sponsorships are required to return to Singapore after their overseas stints, thereby providing a guaranteed pool of research talent in Singapore to bolster local bioinformatics R&D. In addition, we warmly welcome bioinformaticists and computational biologists to Singapore—http://www.comp.nus.edu.sg/~wongls/openings.html lists some of the opportunities in Singapore.
