# Correction: Brief Overview of Bioinformatics Activities in Singapore

**DOI:** 10.1371/annotation/5d07cf0b-75f8-4452-a932-46574f5faf3f

**Published:** 2009-11-04

**Authors:** Frank Eisenhaber, Chee-Keong Kwoh, See-Kiong Ng, Wing-Kin Sung, Limsoon Wong

The fourth author's name was spelled incorrectly. The correct name is: Wing-Kin Sung.

